# The Global Lung Function Initiative 2012 Equations Are as Well-Suited as Local Population Derived Equations to a Sample of Healthy Professional Firefighters

**DOI:** 10.1155/2017/6327180

**Published:** 2017-05-25

**Authors:** Flynn Slattery, Tjard Schermer, Adrian Esterman, Kylie Johnston, Alan Crockett

**Affiliations:** ^1^Alliance for Research in Exercise, Nutrition and Activity (ARENA), Sansom Institute, School of Health Sciences, University of South Australia, Adelaide, SA, Australia; ^2^Department of Primary and Community Care, Radboud University Medical Centre, Nijmegen, Netherlands; ^3^School of Nursing and Midwifery, University of South Australia, Adelaide, SA, Australia; ^4^School of Health Sciences, Sansom Institute for Health Research, University of South Australia, Adelaide, SA, Australia

## Abstract

**Background and Objective:**

We aimed to assess the validity of using the Global Lung Function Initiative's (GLI) 2012 equations to interpret lung function data in a healthy workforce of South Australian Metropolitan Fire Service (SAMFS) personnel.

**Methods:**

Spirometry data from 212 healthy, nonsmoking SAMFS firefighters were collected and predicted normal values were calculated using both the GLI and local population derived (Gore) equations for forced expiratory volume in one second (FEV_1_), forced vital capacity (FVC), and FEV_1_/FVC. Two-tailed paired sample Student's *t*-tests, Bland-Altman assessments of agreement, and *z*-scores were used to compare the two prediction methods.

**Results:**

The equations showed good agreement for mean predicted FEV_1_, FVC, and FEV_1_/FVC. Mean *z*-scores were similar for FEV_1_ and FVC, although not FEV_1_/FVC, but greater than 0.5. Differences between the calculated lower limits of normal (LLN) were significant (*p* < 0.01), clinically meaningful, and resulted in an 8% difference in classification of abnormality using the FEV_1_/FVC ratio.

**Conclusions:**

The GLI equations predicted similar lung function as population-specific equations and resulted in a lower incidence of obstruction in this sample of healthy SAMFS firefighters. Further, interpretation of spirometry data as abnormal should be based on both an FEV_1_ and FEV_1_/FVC ratio < LLN.

## 1. Introduction

Firefighters' risk of developing chronic respiratory diseases is well known and no better exemplified than by the marked deterioration in lung function of first responders to the 9/11 disaster in New York [1]. The US-based National Fire Protection Association (NFPA) now recommends that spirometry be performed on an annual basis [2].

The interpretation of the results of spirometry rely, in part, on their comparison to a reference standard derived from normative data obtained in a healthy population. The 2005 American Thoracic Society and European Respiratory Society (ATS/ERS) statement on spirometry recommended the use of population-specific predicted normal equations [3], which should be updated approximately every ten years to reflect the changes that are likely to occur in anthropometric and ethnic characteristics (e.g., changes in mean heights for a given age over time). This is logistically difficult to do in every population. The Global Lung Function Initiative (GLI) 2012 therefore developed a new set of multiethnic predicted normal equations [4, 5] using the pooled resources of 26 countries and data from more than 74000 subjects. They have been evaluated and shown to be well matched to some adult populations (in Australasia [6] and Europe [7, 8]) but not others, such as Finland [9] and Sweden [10], where local population-specific reference values may be more relevant. Therefore, care needs to be taken when recommending whether GLI equations should be implemented in any particular population or laboratory.

This issue must be considered when interpreting the lung function of professional firefighters. We have previously shown larger forced expiratory volumes in one second (FEV_1_) and, in particular forced vital capacities (FVC), in South Australian Metropolitan firefighters compared to age-matched controls, in both the entire sample and the majority who have no history of doctor-diagnosed lung disease [11]. Larger values may be attributable to a “healthy worker effect” [12], as well as the relatively high standard of physical fitness required for entry into the fire service. Selection of the most appropriate reference equations relative to this cohort is important in both the surveillance of serving firefighters and the assessment of potential recruits.

The purpose of this paper is to determine if the GLI equations are well matched to this healthy workforce, in light of their relatively large FVCs, compared to reference equations that were derived from the local population [13] (and not included in the GLI pool of data). We hypothesised that there would be no difference in the number of fire fighters who would be classified as having abnormal results (lung function less than the lower limit of normal (LLN)) between the two equations.

## 2. Materials and Methods

### 2.1. Study Participants and Data Collection

We used spirometric data from full-time South Australian Metropolitan Fire Service (SAMFS) firefighters collected between June 2014 and April 2015 using a pneumotachograph-based spirometer (Masterscreen™ PFT system, CareFusion, Yorba Linda, CA). Spirometric data were collected as part of the ongoing longitudinal surveillance of lung function and respiratory health in the SAMFS, which commenced in 2007. All spirometry was performed prebronchodilator and in accordance with ATS/ERS guidelines [14]. Age was calculated to at least one decimal point as the difference between date of birth and date of examination. Participants provided information on medical and smoking history by written questionnaire following spirometry. Only never-smokers and firefighters with no history of doctor-diagnosed asthma or lung disease, based on questionnaire responses, were included in this analysis. Further details of procedures and equipment used in data collection have been previously described [11, 15, 16]. Calibration was performed on a daily basis using a three-litre syringe while zero flow was set immediately before each measurement. Data collection was funded by the SAMFS and ethical approval was obtained from the University of South Australia Human Research Ethics Committee (0000032662).

### 2.2. Reference Equations

Predicted normal values for FEV_1_, FVC, and the FEV_1_/FVC ratio were calculated for each subject using two different sets of equations: firstly, using prediction equations derived from a random sample of the South Australian population that also used a pneumotachograph-based spirometer (Gore) [13] and, secondly, using prediction equations from the Global Lung Function Initiative (GLI) [4], following the specific instructions. Individual *z*-scores were calculated by subtracting the predicted value from the measured value and dividing by the standard deviation. The individual LLN was statistically defined by the lower fifth percentile (i.e., *z*-score = −1.645).

### 2.3. Data Analysis

Data were checked for normal distribution and two-tailed paired samples Student's* t*-tests or Wilcoxon Signed Ranks Tests were used to compare the means of predicted lung function and the LLNs, as well as mean *z*-scores. Independent samples Student's *t-*tests were used to compare included firefighters to those excluded based on medical history. The *z*-score is a standardised measure of the position of a measurement within the distribution of the population from which the reference values are derived and takes into account age and height-related variability. We follow Hall and colleagues in defining the minimum physiologically relevant difference to be 0.5 *z*-scores [6]. A significance level of *p* = 0.01 was set for all tests to allow for multiple testing. Bland-Altman's 95% limits of agreement analysis (LoA) were used to quantify the difference and random error between the two equations. Bland-Altman plots provide information on how the difference between the two equations changes as the scale increases/decreases [17]. Limits of agreement were defined as mean difference ± 1.96 SD. Good agreement was defined by the LoA being less than ATS/ERS standard of acceptable repeatability (0.15 L) [14]. Data were analysed using SPSS®, version 22.0.0 for Windows, PC (IBM, Chicago, IL, USA).

## 3. Results

### 3.1. Characteristics of Study Population

From spirometry measures collected in 409 full-time firefighters, 212 participants were included in this analysis. The five full-time female firefighters were excluded from the analyses as well as a further two males (due to incomplete spirometry data). Twenty-one firefighters were excluded for having incomplete information on smoking status, along with 13 current smokers and 87 former smokers. A further 69 firefighters were excluded based on having a history of doctor-confirmed asthma or respiratory disease. The mean age (SD) of the included participants was 46.4 (8.7) years, mean height was 181.1 (6.2) cm, and mean body mass was 89.6 (12.6) kg. Measured spirometric values, predicted values, and LLNs calculated using both reference equations are shown in Table 1. Comparing included and excluded firefighters (see Supplementary Table 1 in Supplementary Material available online at https://doi.org/10.1155/2017/6327180), measured FEV_1_ and FVC and mean *z*-scores using Gore for FEV_1_ and FVC were significantly lower in the excluded firefighters (*p* < 0.01).

### 3.2. Differences between Prediction Equations

**Figure 1:**
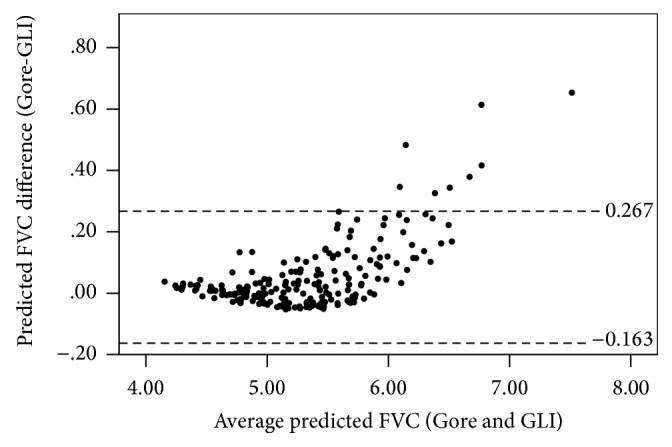
Differences of predicted FVC for Gore compared with GLI illustrating the small systematic difference at higher values (*N* = 212).

The mean predicted values and LLNs calculated using the GLI equations were significantly (*p* < 0.01) different from those produced using the Gore equations, excluding mean predicted FEV_1_/FVC (Table 1). Mean differences (95% confidence interval of the difference [CI]) ± LoA (Gore relative to GLI) were 20 (16–25) ± 65 mL and 52 (37–66) ± 215 mL for predicted FEV_1_ and FVC, respectively, while there was virtually no difference between the two predicted FEV_1_/FVC ratios. Bland-Altman plots of predicted FVC revealed a small systematic difference at high FVCs (Figure 1), while no clinically relevant systematic differences were observed for FEV_1_ or FEV_1_/FVC (data not shown).

**Figure 2:**
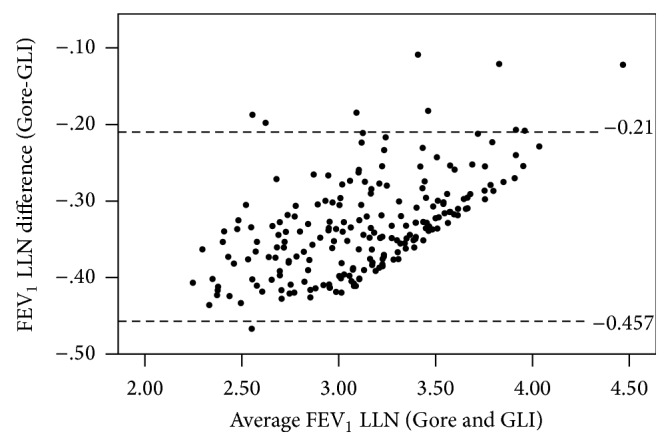
Differences of FEV_1_ LLN for Gore compared with GLI illustrating the lower LLN for GLI, particularly for lower values (*N* = 212).

**Figure 3:**
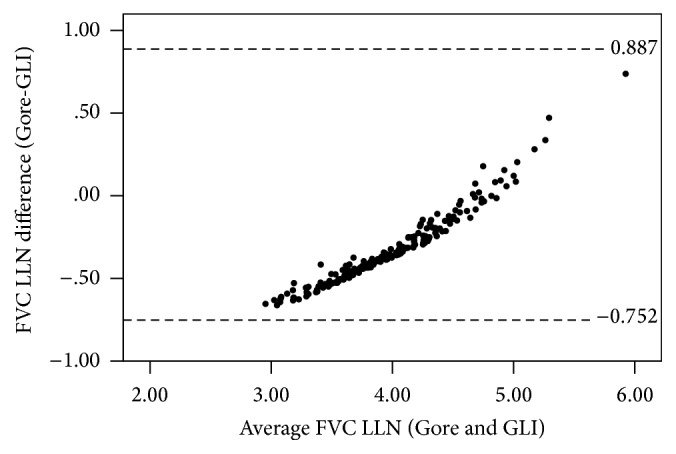
Differences between FVC LLN for Gore compared with GLI illustrating the lower LLN for GLI, particularly for lower values (*N* = 212).

**Figure 4:**
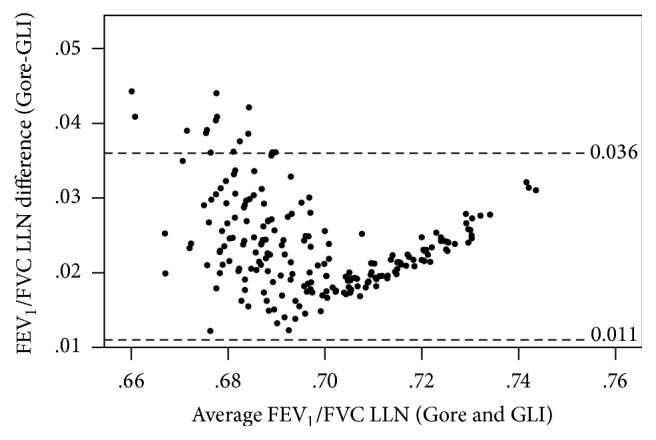
Differences between the LLN FEV_1_/FVC ratio for Gore compared with GLI illustrating the systematic difference at higher values (*N* = 212).

There were more substantial differences in the lower limits of normal with mean differences (95% CI) ± LoA (Gore relative to GLI) of −334 (−342–−325) ± 124 mL and −332 (−361–−303) ± 420 mL for FEV_1_ and FVC, respectively, with a mean difference of 0.024 (0.023–0.025) ± 0.012 for the FEV_1_/FVC ratio. Bland-Altman plots showed some systematic differences at lower values for the FEV_1_ LLN and FVC LLN (Figures 2 and 3) and some systematic differences at higher values for the FEV_1_/FVC ratio LLN (Figure 4). The number of firefighters below the LLN (*z*-score < −1.645) for FEV_1_ was one (<1%) and three (1.4%) (Gore and GLI, resp.) while there were no firefighters below the LLN for FVC. Further, 47 (22.2%) and 30 (14.2%) were below the FEV_1_/FVC LLN for Gore and GLI, respectively: a difference of 8%.

Amongst all firefighters, there was a statistically significant (*p* < 0.01) difference between mean *z*-scores produced by each equation for the FEV_1_/FVC ratio, but not FEV_1_ or FVC (Table 2). When categorised by age, younger firefighters tended to have higher FEV_1_ and FVC *z*-scores with Gore relative to GLI, with this pattern reversing as age increased. Mean GLI FEV_1_/FVC ratio *z*-scores were generally closer to zero amongst all age categories than those produced with Gore.

## 4. Discussion

This analysis demonstrated that the GLI equations are as well-suited to a sample of healthy professional firefighters, who typically have above-average lung function, as the population-specific Gore equations.

The two equations in our study showed good agreement for mean predicted FEV_1_ and FEV_1_/FVC, but not for FVC, which was of clinical importance. There was also a significant difference between mean *z*-scores for FEV_1_/FVC, but not FEV_1_ and FVC. Hall and colleagues previously determined that GLI equations are well matched to Australasian spirometry [6], reporting mean *z*-scores (SD) of 0.23 (1.00) for FEV_1_, 0.23 (1.00) for FVC, and −0.03 (0.87) for FEV_1_/FVC using the GLI equations. Observed FEV_1_ and FVC *z*-score means in our sample were both greater than those observed by Hall and colleagues, as well as the minimum physiologically relevant difference of 0.5 *z*-scores. These higher values may be partly attributable to a healthy worker effect or to the preemployment selection process. Potential recruits with low lung function may be excluded directly as part of their prehire mandatory medical evaluations, while the intense prehire physical fitness evaluations of simulated firefighting tasks may naturally select those with above-average lung function. A possible explanation for the low FEV_1_/FVC ratios (*z*-score means <−0.7 for both equations) in the presence of above-average FEV_1_ may involve the concept of airway/parenchymal dysanapsis, whereby an individual may have comparatively large lungs (which determines FVC) without a correspondingly large airway diameter (which determines FEV_1_) [18, 19], although why this phenomenon would feature so prominently in this population is unclear.

These analyses showed considerable differences between the subsequent LLNs, which were clinically meaningful, given their recommended use in detecting abnormality [3]. The impact of switching reference equation on the incidence of airflow obstruction has been investigated, with both Quanjer et al. and Brazzale et al. observing minimal differences when comparing the GLI to the European Community of Steel and Coal (ECSC) and The Third National Health and Nutrition Examination Survey (NHANES III) equations [5, 20]. Hulo et al., however, observed more considerable differences when comparing the GLI to the ECSC equations [8]. By definition, five per cent of a healthy population sample would be expected to be below the LLN (lower 5th percentile). In this firefighter cohort, rates of FEV_1_/FVC less than the LLN (indicative of obstruction) were higher than this, as well as those reported by Backman et al. [10] (2.7%) and Hulo et al. [8] (7.2%), yet lower than both Brazzale et al. (27.4%) [20] and Quanjer et al. (34.5%) [5], when using the GLI equations. However, when interpretations are made using clinically important airflow limitation (when both the FEV_1_/FVC and the FEV_1_ are below their LLNs) the rates of abnormality were greatly reduced to ≤2% for both equations. While some organisations such as the British Thoracic Society and the Global Initiative for Chronic Obstructive Lung Disease advocate the use of evaluating FEV_1_ with the FEV_1_/FVC ratio to grade the severity of obstruction [21, 22], it is of particular importance in selected (healthy) populations with large FVCs, to reduce the likelihood of misclassification. Such misclassification has important practical implications for firefighters, beyond the obvious detection of disease or abnormality, given the ongoing recommendation from firefighting organisations that prehire medicals determine abnormal spirometry based on a fixed cut-off of the FEV_1_/FVC ratio alone [2, 23].

The NFPA recommends annual spirometric assessment of firefighter lung function, with interpretations based on expressing lung function as a percentage of predicted normal, adjusted for age, height, gender, and ethnicity [2]. Such interpretations may systematically misclassify diseased firefighters whose lung function was greatly above normal in the first instance. A more valid means of examining lung function in a population like this is to examine the annual rate of change for each individual and compare this to an established limit of normal longitudinal decline [24]. This is the intention of our surveillance program, and preliminary results have previously been reported [15]. Longitudinal surveillance may also reduce the misclassification of those whose lung function lies close to the LLN or upper limit of normal, given that such classifications can change over follow-up [25].

### 4.1. Limitations of This Study

A limitation of this analysis is that it was not known whether any of the firefighters truly had clinically diagnosed or undiagnosed obstructive lung disease, given that this information was self-reported. Although FEV_1_ was normal, disease may still have been present if participants had abnormally large FEV_1_ at the beginning of their careers.

The present study used and discussed the validity of the FEV_1_/FVC ratio and its implications for assessing obstruction in firefighters. The ATS/ERS however define obstruction as a reduced FEV_1_ to vital capacity (VC) ratio, below the 5th percentile of the predicted value [3]. As slow VC is expected to be greater than FVC [26], use of the FEV_1_/VC ratio could potentially increase the likelihood of misclassified obstruction in a population with proportionally large lungs relative to airway diameter.

At the time of the study, the SAMFS maintained a workforce of 861 full-time firefighters, 409 of whom voluntarily participated (47.5%). The main reason for nonparticipation was for logistical reasons, as a large portion of nonparticipating firefighters were either in nonmetropolitan areas or not present during scheduled lung function testing at a given station; many SAMFS firefighters hold positions unattached to a particular station and frequently move between locations. While privacy and anonymity were ensured, some firefighters with respiratory symptoms or asthma or who smoked may have chosen not to participate, possibly contributing to the above-average lung function observed in this study. Those who did participate may also have denied certain information.

A further limitation of the study was the relatively narrow age range of the men. The LLN for the Gore equations was calculated by subtracting the measured value from the predicted value while GLI equations use the lambda-mu-sigma method (to account for the larger variation seen in older adults). The differences in the subsequent LLNs are accentuated when many older adults are included in the sample. However, firefighters are usually less than the age of 60 and so the results are no less valid for this population.

## 5. Conclusions

The GLI equations predicted similar lung function as population-specific equations and resulted in a lower incidence of obstruction in this sample of healthy SAMFS firefighters. Identification of abnormal spirometry should rely on interpretation of both the FEV_1_/FVC ratio and the FEV_1_ value in relation to the LLN.

## Supplementary Material

Supplementary Table 1: Comparison of included healthy SAMFS firefighters and excluded firefighters with a history of doctor-confirmed asthma or lung disease. Values are means (standard deviation). Lung function measured pre-bronchodilator.

## Figures and Tables

**Table 1:** Measured and predicted lung function values using Gore and GLI equations. Participants are never-smokers with no history of doctor-confirmed asthma or lung disease. Values are means (standard deviation) (*N *= 212).

	Measured^*∗*^	Predicted		LLN	
		Gore	GLI	Gore	GLI
FEV_1_ (L)	4.52 (0.67)	4.22 (0.42)	4.20 (0.45)^$^	2.95 (0.42)	3.29 (0.39)^$^
FVC (L)	6.05 (0.82)	5.35 (0.61)	5.30 (0.54)^$^	3.81 (0.61)	4.14 (0.40)^$^
FEV_1_/FVC ratio	0.75 (0.06)	0.80 (0.02)	0.80 (0.02)	0.71 (0.02)	0.69 (0.02)^$^

SAMFS = South Australian Metropolitan Fire Service; LLN = lower limit of normal; FEV_1_ = forced expiratory volume in 1 second; FVC = forced vital capacity; FEV_1_/FVC ratio = forced expiratory volume in 1 second to forced vital capacity ratio. ^*∗*^Prebronchodilator values. ^$^Statistically significant difference between Gore and GLI with Student's *t*-test (*p* < 0.01).

**Table 2:** Average *z*-scores by age category. Participants are never-smokers with no history of doctor-confirmed asthma or lung disease. Values are mean (standard deviation) (*N *= 212).

Age groups	Equation	FEV_1_	FVC	FEV_1_/FVC ratio
<30 (*n* = 7)	Gore	0.64 (0.85)	0.96 (0.62)	−0.77 (1.61)
	GLI	−0.16 (1.05)	0.41 (0.73)	−0.88 (1.18)
30–39 (*n* = 45)	Gore	0.89 (0.76)^$^	1.30 (0.71)^$^	−0.96 (1.20)
	GLI	0.35 (0.99)	0.79 (0.69)	−0.83 (0.92)
40–49 (*n* = 67)	Gore	0.91 (0.80)^$^	1.39 (0.78)^$^	−1.09 (1.03)^$^
	GLI	0.65 (1.01)	1.00 (0.72)	−0.67 (0.85)
50–59 (*n* = 86)	Gore	0.32 (0.88)^$^	0.91 (0.99)^$^	−1.49 (1.09)^$^
	GLI	0.72 (1.03)	1.26 (1.05)	−0.74 (0.83)
≥60 (*n* = 7)	Gore	0.18 (0.75)^#^	0.66 (0.67)^#^	−1.30 (0.66)^#^
	GLI	1.26 (1.03)	1.77 (1.06)	−0.42 (0.53)
All (*n* = 212)	Gore	0.63 (0.87)	1.14 (0.87)	−1.22 (1.12)^$^
	GLI	0.61 (1.03)	1.07 (0.90)	−0.73 (0.86)

SAMFS = South Australian Metropolitan Fire Service; LLN = lower limit of normal; FEV_1_ = forced expiratory volume in 1 second; FVC = forced vital capacity; FEV_1_/FVC ratio = forced expiratory volume in 1 second to forced vital capacity ratio. ^$^Statistically significant difference between Gore and GLI with Student's *t*-test (*p* < 0.01). ^#^Statistically significant difference between Gore and GLI with Wilcoxon Signed Ranks test (*p* < 0.01).

## References

[1] Aldrich T. K., Gustave J., Hall C. B. (2010). Lung function in rescue workers at the world trade center after 7 years. *New England Journal of Medicine*.

[2] National Fire Protection Association 1582 (2013). *Standard on Comprehensive Occupational Medical Programs for Fire Departments*.

[3] Pellegrino R., Viegi G., Brusasco V. (2005). Interpretative strategies for lung function tests. *European Respiratory Journal*.

[4] Quanjer P. H., Stanojevic S., Cole T. J. (2012). Multi-ethnic reference values for spirometry for the 3-95-yr age range: the global lung function 2012 equations. *European Respiratory Journal*.

[5] Quanjer P. H., Brazzale D. J., Boros P. W., Pretto J. J. (2013). Implications of adopting the Global Lungs Initiative 2012 all-age reference equations for spirometry. *The European Respiratory Journal*.

[6] Hall G. L., Thompson B. R., Stanojevic S. (2012). The Global Lung Initiative 2012 reference values reflect contemporary Australasian spirometry. *Respirology*.

[7] Hüls A., Krämer U., Stolz S. (2016). Applicability of the global lung initiative 2012 reference values for spirometry for longitudinal data of elderly women. *PLoS ONE*.

[8] Hulo S., de Broucker V., Giovannelli J. (2016). Global lung function initiative reference equations better describe a middle-aged, healthy french population than the european community for steel and coal values. *European Respiratory Journal*.

[9] Kainu K., Timonen J., Toikka B. (2015). Reference values of spirometry for finnish adults. *Clinical Physiology and Functional Imaging*.

[10] Backman H., Lindberg A., Sovijarvi A., Larsson K., Lundback B., Ronmark E. (2015). Evaluation of the global lung function initiative 2012 reference values for spirometry in a Swedish population sample. *BMC Pulmonary Medicine*.

[11] Schermer T. R., Malbon T., Morgan M. (2010). Lung function and health status in metropolitan fire-fighters compared to general population controls. *International Archives of Occupational and Environmental Health*.

[12] Li C.-Y., Sung F.-C. (1999). A review of the healthy worker effect in occupational epidemiology. *Occupational Medicine*.

[13] Gore C., Crockett A., Pederson D., Booth M., Bauman A., Owen N. (1995). Spirometric standards for healthy adult lifetime nonsmokers in Australia. *European Respiratory Journal*.

[14] Miller M. R., Hankinson J., Brusasco V. (2005). Standardisation of spirometry. *European Respiratory Journal*.

[15] Schermer T. R., Malbon W., Adams R., Morgan M., Smith M., Crockett A. J. (2013). Change in lung function over time in male metropolitan firefighters and general population controls: a 3-year follow-up study. *Journal of Occupational Health*.

[16] Schermer T. R., Malbon W., Newbury W. (2010). Spirometry and impulse oscillometry (IOS) for detection of respiratory abnormalities in metropolitan firefighters. *Respirology*.

[17] Bland J. M., Altman D. G. (2010). Statistical methods for assessing agreement between two methods of clinical measurement. *International Journal of Nursing Studies*.

[18] Martin T. R., Feldman H. A., Fredberg J. J., Castile R. G., Mead J., Wohl M. (1988). Relationship between maximal expiratory flows and lung volumes in growing humans. *Journal of Applied Physiology*.

[19] Mead J. (1980). Dysanapsis in normal lungs assessed by the relationship between maximal flow, static recoil, and vital capacity. *The American Review of Respiratory Disease*.

[20] Brazzale D. J., Hall G. L., Pretto J. J. (2013). Effects of adopting the new global lung function initiative 2012 reference equations on the interpretation of spirometry. *Respiration*.

[21] National Clinical Guideline Centre—Acute and Chronic Conditions (2010). *Chronic obstructive pulmonary disease: Management of chronic obstructive pulmonary disease in adults in primary and secondary care*.

[22] GOLD (2017). Global strategy for the diagnosis, management, and prevention of chronic obstructive pulmonary disease. *Global Initiative for Chronic Obstructive Lung Disease (GOLD)*.

[23] (2002). *AFAC Guidelines for Health and Fitness Monitoring of Australasian Fire and Emergency Service Workers.*.

[24] Hnizdo E. (2012). The value of periodic spirometry for early recognition of long-term excessive lung function decline in individuals. *Journal of Occupational and Environmental Medicine*.

[25] Schermer T. R., Robberts B., Crockett A. J. (2016). Should the diagnosis of COPD be based on a single spirometry test?. *NPJ Primary Care Respiratory Medicine*.

[26] Constán E., Medina J., Silvestre A., Alvarez I., Olivas R. (2005). Difference between the slow vital capacity and forced vital capacity: predictor of hyperinflation in patients with airflow obstruction. *The Internet Journal of Pulmonary Medicine*.

