# Supplementary material for: The Global Lung Function Initiative 2012 Equations Are as Well-Suited as Local Population Derived Equations to a Sample of Healthy Professional Firefighters
Source: Can Respir J. 2017 May 25;2017:6327180. doi: 10.1155/2017/6327180 (PMC5463135; doi:10.1155/2017/6327180)
Supplement: Supplementary file 1 — Supplementary Table 1: Comparison of included healthy SAMFS firefighters and excluded firefighters with a history of doctor-confirmed asthma or lung disease. Values are means (standard deviation). Lung function measured pre-bronchodilator. [file 6327180.f1.docx]

SUPPLEMENTARY TABLE 1: Comparison of included healthy SAMFS firefighters and excluded firefighters with a history of doctor-confirmed asthma or lung disease. Values are means (standard deviation). Lung function measured pre-bronchodilator.

|  | All firefighters  (n=402) | Included firefighters  (n=212) | Excluded firefighters  (n=190) |
| --- | --- | --- | --- |
| Age (y) | 47.4 (8.7) | 46.4 (8.7) | 48.6 (8.6) |
| Weight (kg) | 90.4 (12.6) | 89.6 (12.6) | 91.2 (12.7) |
| Height (cm) | 180.5 (6.2) | 181.1 (6.2) | 179.8 (6.1) |
| FEV_1_ (L) | 4.40 (0.70) | 4.52 (0.67) | 4.27 (0.72)^$^ |
| z-score (Gore) | 0.48 (0.91) | 0.63 (0.87) | 0.32 (0.93)^$^ |
| z-score (GLI) | 0.51 (1.04) | 0.61 (1.03) | 0.41 (1.04) |
| FVC (L) | 5.93 (0.85) | 6.05 (0.82) | 5.79 (0.86)^$^ |
| z-score (Gore) | 1.01 (0.91) | 1.14 (0.87) | 0.87 (0.92)^$^ |
| z-score (GLI) | 1.01 (0.95) | 1.07 (0.90) | 0.95 (1.00) |
| FEV_1_/FVC ratio | 0.74 (0.06) | 0.75 (0.06) | 0.74 (0.06) |
| z-score (Gore) | -1.29 (1.17) | -1.22 (1.12) | -1.37 (1.23) |
| z-score (GLI) | -0.76 (0.87) | -0.73 (0.86) | -0.79 (0.89) |
| SAMFS = South Australian Metropolitan Fire Service; LLN = lower limit of normal; FEV_1_ = forced expiratory volume in 1 second; FVC = forced vital capacity; FEV_1_/FVC ratio = forced expiratory volume in 1 second to forced vital capacity ratio. ^$^Statistically significant difference between Included and excluded firefighters with Student’s t-test (*p* < 0.01). | | | |
